# Der Stellenwert von Leitlinien in der Zahnheilkunde und in der zahnmedizinischen Ausbildung

**DOI:** 10.1007/s00103-023-03798-x

**Published:** 2023-11-14

**Authors:** Sebastian Krohn, Ina B. Kopp, Peter Proff

**Affiliations:** 1 Poliklinik für Kieferorthopädie, Universitätsklinikum Regensburg, Franz-Josef-Strauß-Allee 11, 93053 Regensburg, Deutschland; 2 Arbeitsgemeinschaft der Wissenschaftlichen Medizinischen Fachgesellschaften e. V. (AWMF), Berlin, Deutschland

**Keywords:** Leitlinien, Evidenzbasierte Zahnmedizin, Konsensus, Arbeitsgemeinschaft Wissenschaftlicher Medizinischer Fachgesellschaften (AWMF), Zahnmedizinische Lehre, Guidelines, Evidence-based dentistry, Consensus, Association of Scientific Medical Societies (AWMF), Dental education

## Abstract

Im Rahmen von qualitätssichernden Maßnahmen spielen evidenzbasierte Handlungsempfehlungen in der Zahn- und Humanmedizin eine zunehmend bedeutendere Rolle. Die von Expertengremien methodenkritisch evaluierten Wissenschaftserkenntnisse werden dabei zu einfach verständlichen Leitlinien zusammengefasst. Entsprechend der Konsens- und Evidenzgewichtung des Erstellungsprozesses werden die Leitlinien in verschiedenen Entwicklungsstufen qualitativ bewertet. Seit der Gründung in den 1960er-Jahren erfolgen die Publikation von Leitlinien und die Koordination der Leitlinienerstellung durch die Arbeitsgemeinschaft der Wissenschaftlichen Medizinischen Fachgesellschaften (AWMF). Zum aktuellen Zeitpunkt sind 44 zahnmedizinische Leitlinien verfügbar, die zum größten Teil mit der höchsten Entwicklungsstufe S3 bewertet sind. Dadurch sind Handlungskorridore für eine Vielzahl von Behandlungsmaßnahmen für zahnärztliches Personal der universitären Standorte und Praxen definiert, deren Implementierung in den klinischen Alltag wünschenswert ist. Mangelnde Akzeptanz von Leitlinien und ein hoher Ressourcenaufwand bei deren Erstellung sind wesentliche Limitationen im Ausbau der evidenzbasierten Zahnmedizin. Diese könnten durch Einführung der wissenschaftlichen Grundausbildung innerhalb des Zahnmedizinstudiums und verstärkte Förderung des wissenschaftlichen Nachwuchses überwunden werden, um hohe Behandlungsqualität und Ökonomie in der Zahnheilkunde auch zukünftig zu gewährleisten. Leitlinien können die zahnmedizinische Ausbildung unterstützen, indem sie Studierenden wissenschaftlich abgesicherte Handlungsschablonen bieten und den Lehrenden helfen, den hohen Anforderungen im Rahmen von praktischen Kursen gerecht zu werden.

## Abstract (en)

Evidence-based treatment recommendations are gaining importance within the framework of both medical and dental quality management systems. The scientific findings, which have been evaluated critically by expert committees with regard to their methodological quality, are summarized in easy-to-understand guidelines. All guidelines are evaluated qualitatively in accordance with a balance between consensus and evidence during the drafting process regarding their stages of scientific development. The publication of guidelines and the coordination of guideline development has been carried out by the Association of the Scientific Medical Societies in Germany (AWMF) since its foundation in the 1960s. Forty-four dental guidelines are currently available, which are mostly rated at the highest level (S3) of scientific development. Therefore, recommendations for various treatment protocols are defined for both dental staff at university sites or practices and the implementation of these guidelines into the daily clinical routine is desirable. Poor acceptance and adverse resource requirements are major limitations of the establishment of guidelines with regard to the expansion of evidence-based dentistry. However, these limitations might be overcome by the introduction of basic scientific training within dental universities and increased funding of young researchers in order to ensure high treatment quality and economy in dentistry in the future. Guidelines can facilitate education by providing scientifically validated procedural templates to dental students and assisting educators in meeting the requirements of practical skills.

## Einleitung

Qualitätssichernde Maßnahmen dienen der Optimierung von Prozessen im Hinblick auf Sicherheit, Effizienz und Wirtschaftlichkeit [1]. Auch in der Human- und Zahnmedizin dienen systematisch entwickelte Handlungsempfehlungen der Entscheidungsfindung, was zu qualitativ hochwertigen und medizinisch angemessenen Behandlungsmaßnahmen führen soll [2]. Um dies zu gewährleisten, sollten klinische Handlungsempfehlungen durch einen zeitnahen Transfer aktueller Forschungserkenntnisse aus der Wissenschaft gekennzeichnet sein [3]. Gleichzeitig sollten die resultierenden Empfehlungen im Sinne dynamischer Prozesse in regelmäßigen Zyklen auf Aktualität und klinischen Wert überprüft werden [1].

Allerdings ist die Bewertung der Qualität wissenschaftlicher Erkenntnisse häufig keine leichte Aufgabe. Mit dem Aufkommen der evidenzbasierten Medizin in den 1990er-Jahren wurde das Ziel verfolgt, publizierte Studiendaten anhand von Evidenzhierarchien zu evaluieren und somit für diagnostische und therapeutische Anwendungen zu gewichten [4]. In der evidenzbasierten Zahnheilkunde werden laut Definition der American Dental Association (ADA) systematisch evaluierte, klinisch relevante wissenschaftliche Erkenntnisse, die den zahnärztlichen und allgemeinmedizinischen Zustand sowie die Vorgeschichte von PatientInnen berücksichtigen, sorgfältig integriert. Dies soll in Anbetracht des klinischen Fachwissens der Behandelnden und der Behandlungsbedürfnisse und -präferenzen der PatientInnen erfolgen [2]. Der Begriff Evidenz beinhaltet die Anwendung epidemiologischer, biometrischer und statistischer Methoden, um professionsbezogene Leistungsniveaus zu definieren und durch die beste verfügbare wissenschaftliche Datenlage zu untermauern [4]. Neben der Bewertung der ärztlichen Qualität sollten Behandlungsmaßnahmen allerdings auch an patientenbezogenen Parametern, wie zum Beispiel Lebensqualität und Zufriedenheit, gemessen werden und darüber hinaus ökonomisch sein [5].

## Leitlinien in der Medizin: Historie, Implementierung und Bewertung

### Historie der medizinischen Handlungsempfehlungen.

Erste Bemühungen, eine qualitativ hochwertige und gleichzeitig wirtschaftliche Behandlungsweise in Leitsätzen zu propagieren, gehen auf Professor Friedrich Kraus aus der Berliner Charité im Jahr 1924 zurück [6]. Diese Leitsätze waren auf einen zweckmäßigen Umgang mit Arzneimitteln und Verordnungen sowie auf einen ökonomischen Einsatz von personellen Ressourcen fokussiert, während gleichzeitig an eine fachkompetente Aus- und Weiterbildung von ärztlichem Personal appelliert wurde. Aufgrund demografischer und sozialer Entwicklungen wurde in den letzten Jahrzehnten innerhalb der Medizin erneut eine Handlungsnotwendigkeit aufgezeigt, Richtlinien zu entwerfen, um defizitäre Vorgänge zu vermeiden und notwendige Behandlungsmaßnahmen zu ermöglichen [7]. Im Jahr 1962 wurde daher auf Anregung der Deutschen Gesellschaft für Chirurgie in Frankfurt am Main die Arbeitsgemeinschaft der Wissenschaftlichen Medizinischen Fachgesellschaften (AWMF^1^) gegründet. Während der Gesetzgeber eine stärkere Evidenz von den Leistungserbringern fordert, ermöglicht die AWMF seit den 1990er-Jahren die Koordination der mittlerweile etwa 190 Mitgliedsgesellschaften [4]. Die Hauptaufgabe der AWMF ist die methodische Betreuung der Fachgesellschaften im Hinblick auf die Zusammenstellung von diagnostischen und therapeutischen Empfehlungen sowie die Bereitstellung der Leitlinien der Fachgesellschaften über das qualitätsgesicherte Leitlinienregister „AWMF online“ [8].

### Implementierung von Leitlinien in den klinischen Alltag.

Unter dem Aspekt der Sicherung und Verbesserung der Behandlungsqualität werden im Rahmen eines Leitlinien-Erstellungsprozesses wissenschaftliche Publikationen durch die jeweiligen Fachgesellschaften methodenkritisch evaluiert und zu übersichtlichen und einfach verständlichen sogenannten Leitlinien zusammengefasst [9]. Als wesentliche Kriterien sollten Leitlinien systematisch entwickelte Aussagen beinhalten, die den aktuellsten wissenschaftlichen Kenntnisstand repräsentieren, somit evidente Handlungskorridore für das ärztliche Personal definieren und als Entscheidungshilfen für PatientInnen dienen [4]. Allerdings ersetzen Leitlinien bei Patientenklagen weder Gutachterverfahren [10] noch generieren sie aus juristischer Sicht eine Verbindlichkeit [11]. Daher sind neben fachlicher Qualität und guter Verfügbarkeit der Handlungsempfehlungen auch zusätzliche Strategien der Implementierung in den Alltag erforderlich, um eine Änderung des ärztlichen Routineverhaltens herbeizuführen [12]. Hierzu zählen zum Beispiel die Integration der Handlungsempfehlungen in die medizinischen Dokumentationssysteme sowie die Überwachung der Einhaltung und Wirksamkeit mithilfe von Qualitätsmanagementsystemen [12].

### Bewertung der Qualität von Leitlinien.

Es ist bekannt, dass Leitlinien zu einer Verbesserung der Gesundheitsversorgung beitragen können. Allerdings muss mit zunehmender Anzahl publizierter Leitlinien auch die Frage beantwortet werden, ob eine unabhängige, qualitativ hochwertige Datenakquise durchgeführt wurde [9, 13, 14]. Zu diesem Zweck wurden – entsprechend der Redewendung „guidelines for guidelines“ – wesentliche Anforderungen zur Leitlinienerstellung definiert [7]: Neben der zugrunde liegenden Validität und Evidenz der wissenschaftlichen Datenlage sollten auch Alternativmethoden im Hinblick auf Patientenerwartungen und Kostenverhältnisse abgewogen werden [15]. Empfehlungen sollten in leicht verständlicher Terminologie dokumentiert und in planmäßigen Zeitintervallen revidiert werden [7]. Eine suffiziente Reliabilität der Empfehlungen sollte – unter Berücksichtigung spezifischer Ausnahmefälle – reproduzierbare Ergebnisse bei allen Anwendergruppen gewährleisten und die Sichtweisen aller beteiligten Fachdisziplinen berücksichtigen [15]. In Deutschland erfolgt die öffentliche Bereitstellung der mittlerweile sehr zahlreichen Leitlinien über die AWMF, die auch die hierarchische Untergliederung entsprechend der qualitativen Entwicklungsstufen S1, S2 und S3 etabliert hat [4]. S1-Leitlinien basieren auf informellem Konsens selektierter ExpertInnen, sie unterliegen jedoch noch keinem systematischen Evidenz-Entwicklungsprozess und weisen somit eine geringe Legitimation für die klinische Umsetzung auf [9]. Im Kontrast hierzu werden S2-Leitlinien je nach der Gewichtung zwischen Güte der Konsensfindung (S2*k*) und Evidenzgrad (S2*e*) unterschieden [4]. Bei der S2k-Leitlinie ist die wissenschaftliche Legitimation (Evidenz) eher gering, auch wenn die Legitimation für die Umsetzung über den Konsens des Gremiums hoch ist [9]. Umgekehrt ist es bei der S2e-Leitlinie, denn hier wird durch systematische Literaturrecherche eine hohe wissenschaftliche Legitimation erreicht, auch wenn bei der Legitimation für die Umsetzung ein eher geringer Konsens vorliegt [4]. S3-Leitlinien stellen die höchste Entwicklungsstufe dar. Hier wurde ein hoher Evidenzgrad durch systematische Literaturrecherche gewährleistet, während gleichzeitig ein hoher Konsensgrad bei dem repräsentativen Gremium erzielt wurde [4, 9].

## Erstellung von Leitlinien

### Ressourcenintensität als limitierender Faktor.

Die Erstellung von Leitlinien ist sehr ressourcenintensiv und erfordert daher in den meisten Fällen ein hohes Maß an ehrenamtlichem Engagement [16]. Der hohe Zeitaufwand von etwa 2–3 Jahren, der bei der S3-Leitlinienerstellung typischerweise zum Tragen kommt, um die evidenzbasierte, interdisziplinär konsentierte formale Literaturanalyse zu gewährleisten, führt dazu, dass die Anzahl der S3-Leitlinien deutlich geringer ausfällt als die der S2-Leitlinien [17]. Da die Qualitätsunterschiede zwischen S3 und S2 größer sind als zwischen S1 und S2 [17], sollte die Entwicklung von S3-Leitlinien trotz der Ressourcenintensität dennoch stärker gefördert werden. Im Rahmen der systematischen Literaturrecherche könnten zukünftige Suchstrategien durch künstliche Intelligenz optimiert werden, um lückenlose, objektive Suchergebnisse bei der Entwicklung der Suchalgorithmen zu gewährleisten.

### Methodik und Mikropolitik als limitierende Faktoren.

Auch bei guter wissenschaftlicher Basis einer Leitlinie wird vermutet, dass starke Vorbehalte hinsichtlich der klinischen Leitlinienimplementierung vorgebracht werden [18]. Eine hohe methodische und fachliche Qualität der Leitlinien ist daher die Grundvoraussetzung, um für Akzeptanz in der klinischen Anwendung der Leitlinien zu sorgen [12]. Darüber hinaus sollte die objektive Benennung von Mitgliedern der Steuerungskomitees erfolgen, wobei alle Ebenen der medizinischen Versorgung bei ausgewogenem Geschlechterverhältnis berücksichtigt werden sollten [8]. Verzerrungen der Objektivität durch Interessenkonflikte oder subjektive Wahrnehmungen einzelner ExpertInnen sollten durch strikte Vermeidung kommerzieller Abhängigkeiten [8] und durch eine qualitativ hochwertige Methodik vermieden werden [19]. Im Hinblick auf die objektive Bewertung der Methodik steht international das Bewertungsinstrument AGREE II (Appraisal of Guidelines for Research and Evaluation) zur Verfügung. Dabei handelt es sich um einen Bewertungsbogen in Form einer Checkliste mit 23 Kriterien die in 6 Domänen gruppiert sind [4]. Anhand dieses objektiven Bewertungsinstruments kann die Qualität einer beliebigen Leitlinie ermittelt werden.

### Begrenzte Gültigkeit als Regulativ.

Der beste Schutz vor den oben genannten Limitationen ist die regelmäßige Überarbeitung der Leitlinien, in regelmäßigem Turnus [20], wobei die Überarbeitung idealerweise von externen Expertengremien durchgeführt werden sollte [19].

## Leitlinien in der Zahnheilkunde

In der Zahnheilkunde sind Entscheidungshilfen auch in Form von Leitlinien zunehmend verbreitet [21]. Vor allem innerhalb der letzten 10 Jahre wurde eine Vielzahl von zahnmedizinischen Leitlinien etabliert (siehe Tab. 1 für aktuell gültige Leitlinien), die im Onlinearchiv der AWMF^2^ ohne Beschränkungen zugänglich sind [22]. Mit der Einführung der Leitlinien durch die Deutsche Gesellschaft für Zahn‑, Mund- und Kieferheilkunde (DGZMK) gemeinsam mit der Zahnärztlichen Zentralstelle Qualitätssicherung (ZZQ) wurden zunächst lediglich 3 Pilotleitlinien erarbeitet. Bei diesen Leitlinien („Fissuren- und Grübchenversiegelung“, „Fluoridierungsmaßnahmen zur Kariesprävention“ und „Operative Entfernung von Weisheitszähnen“) handelte es sich um praxisrelevante, erste Leitlinien in Deutschland, die entsprechend der systematisierten, methodischen Kriterien der AWMF entwickelt wurden [23].

**Table 1:** 

Titel der Leitlinie	Erstellungsjahr	Stand	Gültigkeit
Materialunverträglichkeiten bei dentalen, enossalen Implantaten (S3)	2022	12/2022	12/2027 4
Implantat-Versorgung zur oralen Rehabilitation im Zusammenhang mit Kopf-Hals-Bestrahlung (S3)	2003	12/2022	12/2027 3
Die Behandlung periimplantärer Infektionen an Zahnimplantaten (S3)	2016	12/2022	12/2027 2
Dentale digitale Volumentomographie (S2k)	2009	12/2022	12/2027 1
Zahnimplantate bei Diabetes mellitus (S3)	2016	12/2022	12/2027 5
Wurzelspitzenresektion (S2k)	2007	07/2020	07/2025 14
Instrumentelle zahnärztliche Funktionsanalyse und Kieferrelationsbestimmung (S2k)	2015	07/2022	07/2027 6
Therapie des dentalen Traumas bleibender Zähne (S2k)	2015	03/2022	03/2027 7
Zahnärztliche Behandlungsempfehlungen von Kindern und Erwachsenen vor und nach einer Organtransplantation (S2k)	2021	10/2021	10/2026 10
Ideale Behandlungszeitpunkte kieferorthopädischer Anomalien (S3)	2021	12/2021	12/2026 8
Die Unterkieferprotrusionsschiene (UPS): Anwendung in der zahnärztlichen Schlafmedizin beim Erwachsenen (S1)	2021	11/2021	11/2026 9
Vollkeramische Kronen und Brücken (S3)	2014	03/2021	02/2026 12
Umgang mit zahnmedizinischen Patienten bei Belastung mit Aerosol-übertragbaren Erregern (S1)	2020	02/2022	03/2026 11
Implantatprothetische Versorgung des zahnlosen Oberkiefers (S3)	2013	11/2020	10/2025 14
Die Behandlung von Parodontitis Stadium I bis III	2020	12/2020	11/202513
Implantologische Indikationen für die Anwendung von Knochenersatzmaterialien (S2k)	2011	07/2020	06/2025 15
Dentale Implantate bei Patienten mit Immundefizienz (S3)	2019	07/2019	06/2024 21
Totaler alloplastischer Kiefergelenkersatz (S3)	2020	04/2020	03/2025 16
Diagnostik und Management von Vorläuferläsionen des oralen Plattenepithelkarzinoms in der Zahn‑, Mund- und Kieferheilkunde (S2k)	2010	09/2019	08/2024 18
Ersatz fehlender Zähne mit Verbundbrücken (S3)	2019	07/2019	06/2024 22
Zahnbehandlungsangst beim Erwachsenen (S3)	2019	10/2019	10/2024 17
Subgingivale Instrumentierung (S3)	2019	10/2019	10/2024 16
Okklusale Dysästhesie – Diagnostik und Management (S1)	2019	07/2019	07/2024 20
Operative Entfernung von Weisheitszähnen (S2k) – UPDATE	2006	08/2019	08/2024 19
Odontogene Sinusitis maxillaris (S2k) – UPDATE	2004	06/2019	06/2024 23
Diagnostik und Behandlung des Bruxismus (S3)	2019	05/2019	05/2024 24
Häusliches mechanisches Biofilmmanagement in der Prävention und Therapie der Gingivitis (S3)	2017	11/2018	12/2023 25
Häusliches chemisches Biofilmmanagement in der Prävention und Therapie der Gingivitis (S3)	2017	11/2018	11/2023 26

Aufgrund der entscheidungsleitenden jedoch nicht verpflichtenden Funktion sind Leitlinien im Rahmen der systematischen Qualitätsförderung nicht zwangsläufig ein Bestandteil des klinischen Alltags [7]. Aus Studienergebnissen geht hervor, dass Leitlinien, bei denen fachliche Aspekte im Vergleich zu professionspolitischen Einflüssen im Vordergrund stehen, zumeist akzeptiert werden [23]. Nachdem zunächst Pilotleitlinien von der DGZMK gemeinsam mit der ZZQ und den entsprechenden Fachgesellschaften in mehreren Konsensus-Verfahren entwickelt wurden, sind seit Juli 2008 2 Leitlinienbeauftragte in der DGZMK beschäftigt [21]. In der Humanmedizin ist bereits gut belegt, dass Leitlinien grundsätzlich zu einer verbesserten Gesundheitsversorgung beitragen [9, 13, 14]. Welchen Einfluss zahnmedizinische Leitlinien konkret auf die Ergebnisqualität in der Versorgung haben, ist allerdings noch nicht vollständig geklärt [7]. Aktuell sind 44 zahnmedizinische Leitlinien etabliert, von denen 26 Leitlinien die Klasse S3 aufweisen, während 14 S2k-Leitlinien und 4 S1-Leitlinien verfügbar sind (Stand: 05/23).

Insbesondere Studierende könnten von Leitlinien profitieren, da sie wissenschaftlich abgesicherte Handlungskorridore bieten [24]. Ein „blindes Vertrauen“ auf die Übertragung einer „Handlungsschablone“ auf jeden klinischen Fall sollte jedoch nicht erfolgen, da die Individualität der klinischen Situation einen gewissen Handlungsspielraum erfordern kann [25]. Die zahnärztliche Approbationsordnung (ZApprO), die zuletzt am 18.04.2016 geändert wurde [26], beinhaltet die verbindlichen praktischen und theoretischen Ausbildungsinhalte für Studierende der Vorklinik und Klinik. Ein praktischer Bezug im Sinne der „frühen Praxiskurse“ ermöglicht dabei nach eng verknüpfter praktischer und theoretischer Lehre das frühzeitige Erlernen manueller Fertigkeiten [24]. Damit verbunden ist jedoch auch eine ressourcenintensive Lehre sowie das Risiko von Hygienelücken bei Übungen an PatientInnen, sofern die Prozesskontrolle und die Dokumentation nicht vollumfänglich umgesetzt werden können [27]. Dabei zeigt die aktuelle Generation der Studierenden einen großen Wunsch nach enger praktischer und theoretischer Lehre mit viel Feedback, Mentoring und Coaching [24]. Gleichzeitig klagen Studierende der Zahnmedizin über eine hohe Stressexposition, die durch Umsetzung der ZApprO noch weiter verschärft worden sei [28]. Diese hohen Anforderungen an die Lehrenden, die entsprechend der Approbationsordnung im Rahmen der praktischen Kurse zu erfüllen sind, könnten durch Implementierung von Leitlinien bedarfsgerecht unterstützt werden.

Insbesondere im Hinblick auf Lehrinhalte der praktischen Kurse könnten zahnärztliche Leitlinien eine gemeinsame Grundstruktur für die deutschen Universitätsstandorte darstellen, um eine Vereinheitlichung der Behandlungsqualität zu ermöglichen. Durch den freien Zugriff auf Textpassagen innerhalb des elektronischen Leitlinienregisters der AWMF ist es möglich, wissenschaftlich fundierte Basis-Behandlungsstrategien in die studentische Lehre zu implementieren. Dennoch sollte berücksichtigt werden, dass Leitlinien nicht zu eindimensional entworfen werden, um sowohl qualitativ hochwertige Therapieempfehlungen sowie Handlungskorridore in Studierendenkursen und in der Praxis zu gewährleisten.

## Ausblick

Um die ressourcenintensive Leitlinienarbeit auch zukünftig bewältigen zu können, empfiehlt die AWMF eine wissenschaftliche Grundausbildung bereits im Studium der Medizin und Zahnmedizin zu verankern [29]. Gefordert wird beispielsweise eine wissenschaftliche Grundausbildung zu implementieren [30] sowie die Förderung des wissenschaftlichen Nachwuchses auszubauen [29]. Wie nachhaltig die aktuellen Programme zur Förderung des wissenschaftlichen Nachwuchses für die Leitlinienarbeit tatsächlich sind, kann zum jetzigen Zeitpunkt nur unzureichend bewertet werden [16].

## Footnotes

1 Vorstellung der AWMF: https://www.awmf.org/die-awmf; letzter Zugriff: 18.10.2023.

2 Online-Archiv der AWMF: https://www.awmf.org; letzter Zugriff: 18.10.2023.

## References

[1] Sander T, Müller M-C, Sander T, Müller M-C (2018). Qualitätsmanagement und Wirtschaftlichkeit. Meine Zahnarztpraxis – Ökonomie: Finanz‑, Liquiditäts- und Investitionsplanung, Honorare, Steuern, Gewinn. Springer Berlin Heidelberg.

[2] Chiappelli F (2019). Evidence-based dentistry: two decades and beyond. J Evid Based Dent Pract.

[3] Haynes RB (1993). Some problems in applying evidence in clinical practice. Ann NY Acad Sci.

[4] Hensen P (2023). Versorgungsqualität und Versorgungssicherheit. Qualitätsmanagement im Gesundheitswesen: Grundlagen für Studium und Praxis.

[5] Ollenschläger G, Kirchner H, Fiene M (2001). Leitlinien in der Medizin – scheitern sie an der praktischen Umsetzung?. Internist.

[6] Kraus F (1924). Wie ließe sich die ärztliche Behandlung der Kranken angesichts der jetzigen wirtschaftlichen Notlage der Bevölkerung sparsam und doch sachgemäß gestalten1)?. DMW-Deutsche Medizinische Wochenschrift.

[7] Gerlach FM, Beyer M, Szecsenyi J, Fischer GC (1998). Leitlinien in Klinik und Praxis. Dtsch Arzteblatt-arztliche Mitteilungen-ausgabe A.

[8] Bernardy K, Klose P, Üçeyler N, Kopp I, Häuser W (2008). Methodological fundamentals for the development of the guideline. Schmerz.

[9] Muche-Borowski C, Kopp I (2011). Guideline development. Z Herz‑, Thorax‑, Gefäßchir.

[10] Wienke A (2008) BGH: Leitlinien ersetzen kein Sachverständigengutachten. Gms Mitt Awmf 5: (2008–2005)

[11] Nölling T (2014) Es bleibt dabei: Leitlinien sind nicht rechtlich verbindlich. Gms Mitt Awmf 11: (Doc6)

[12] Selbmann H-K, Kopp I (2005). Implementierung von Leitlinien in den Versorgungsalltag. Die Psychiatr.

[13] Hepner KA, Rowe M, Rost K (2007). The effect of adherence to practice guidelines on depression outcomes. Ann Intern Med.

[14] Thomas LH, Cullum NA, McColl E et al (1996) Guidelines in professions allied to medicine. Cochrane Database Syst Rev 2010:10.1002/14651858.CD000349 PMC13110913 10796531

[15] Lohr KN, Field MJ (1992). Guidelines for clinical practice: from development to use.

[16] Voigt K, Borchers P, Brosse F (2023). Innovationsausschuss des Gemeinsamen Bundesausschusses (G-BA) fördert neue und alte DEGAM-Leitlinien. Z Allg Med.

[17] Hartig S (2005). Evaluation der methodischen Qualität von Leitlinien der medizinischen Versorgung aus dem System der Arbeitsgemeinschaft der Wissenschaftlichen Medizinischen Fachgesellschaften in Deutschland (AWMF).

[18] Aust B, Ohmann C (2000). Previous experiences with the evaluation of guidelines. Disillusionment after an enthusiastic start. Z Arztl Fortbild Qualitatssich.

[19] Mahlknecht P, Glechner A, Gartlehner G (2015). Guideline development: going from evidence to recommendations. Challenges and opportunities—a methodologist’s view. Z Evid Fortbild Qual Gesundhwes.

[20] Pieper D, Ober P, Dressler C, Schmidt S, Mathes T, Becker M (2019). Effizientere Leitlinienerstellung – eine narrative Übersichtsarbeit. Z Evid Fortbild Qual Gesundhwes.

[21] Kirch W, Middeke M, Rychlik RPT (2009). Aspekte der Prävention.

[22] Türp JC (2020). Literaturrecherche in der Zahnmedizin – empfehlenswerte Suchportale. ZWR-Das Dtsch Zahnärzteblatt.

[23] Bergmann-Krauss B, Micheelis W, Szecsenyi J (2010). Akzeptanz von zahnmedizinischen Leitlinien durch Qualitätszirkel. Z Evid Fortbild Qual Gesundhwes.

[24] Springer V (2014). Nachwuchs auf den anspruchsvollen Beruf adäquat vorbereiten. DFZ.

[25] Springer M (2017). Wissenschaftliche Leitlinien: Handlungskorridore mit Nebenausgängen. Junge Zahnarzt.

[26] Bundesgesetzblatt Teil I. Approbationsordnung für Zahnärzte BGBl 2016. (I: 886. In:Zugegriffen:)

[27] Pelka M, Koch A, Kunz B, Petschelt A (2016). Hygiene in der Hochschulzahnmedizin – Teil 1: Risikofaktor Studierende. Krankenhhyg up2date.

[28] Claußen J (2023) Nicht alle Diamanten entstehen unter Druck. DFZ 10.1007/s12614-023-1131-6 PMC10068204 37035011

[29] Treede R-D (2023). Forschungsförderung: Status und zukünftige Herausforderungen aus Sicht der AWMF. Forschungsförderung: Transparente Strukturen gesucht.

[30] AWMF (2008). AWMF-Stellungnahme: Förderung der wissenschaftlichen Medizin schon in der studentischen Ausbildung.

[31] https://www.awmf.org

